# Supplementary material for: Hot spot prediction in protein-protein interactions by an ensemble system
Source: BMC Syst Biol. 2018 Dec 31;12(Suppl 9):132. doi: 10.1186/s12918-018-0665-8 (PMC6311905; doi:10.1186/s12918-018-0665-8)
Supplement: Supplementary file 2 — All relevant MATLAB code. A simple Matlab implement of our predictor. (ZIP 19,742 kb) [file 12918_2018_665_MOESM2_ESM.zip › Additional file 2/Read ASA/ASA website/dbMPIKT/4YFDA.html]

 NetSurfP - Protein Surface Accessibility and Secondary Structure Predictions

  

|  |  |  |
| --- | --- | --- |
|  |  | NetSurfP - Protein Surface Accessibility and Secondary Structure Predictions  Technical University of Denmark |

  


---

```
# For publication of results, please cite:
# A generic method for assignment of reliability scores applied to solvent accessibility predictions.
# Bent Petersen, Thomas Nordahl Petersen, Pernille Andersen, Morten Nielsen and Claus Lundegaard
# BMC Structural Biology 2009, 9:51 doi:10.1186/1472-6807-9-51
#
# Column 1: Class assignment - B for buried or E for Exposed - Threshold: 25% exposure, but not based on RSA
# Column 2: Amino acid
# Column 3: Sequence name
# Column 4: Amino acid number
# Column 5: Relative Surface Accessibility - RSA
# Column 6: Absolute Surface Accessibility
# Column 7: Z-fit score for RSA prediction
# Column 8: Probability for Alpha-Helix
# Column 9: Probability for Beta-strand
# Column 10: Probability for Coil
E E  4YFDA                  1    0.885 154.592   0.949   0.003   0.003   0.994
E T  4YFDA                  2    0.548  76.049   0.225   0.005   0.045   0.951
E P  4YFDA                  3    0.368  52.162   0.509   0.018   0.047   0.935
B P  4YFDA                  4    0.104  14.772  -0.530   0.021   0.279   0.699
E R  4YFDA                  5    0.495 113.401   1.156   0.003   0.718   0.279
B F  4YFDA                  6    0.149  29.864   0.335   0.001   0.900   0.099
E T  4YFDA                  7    0.299  41.513   0.455   0.001   0.900   0.099
E R  4YFDA                  8    0.451 103.325   0.548   0.003   0.718   0.279
E T  4YFDA                  9    0.464  64.343   1.174   0.005   0.336   0.660
B P  4YFDA                 10    0.239  33.928  -0.786   0.005   0.045   0.951
E V  4YFDA                 11    0.608  93.511   0.364   0.004   0.138   0.858
E D  4YFDA                 12    0.611  88.045  -0.037   0.004   0.420   0.576
B Q  4YFDA                 13    0.189  33.809   1.516   0.002   0.816   0.182
E T  4YFDA                 14    0.514  71.250   1.055   0.001   0.959   0.040
B G  4YFDA                 15    0.072   5.643   0.365   0.001   0.900   0.099
E V  4YFDA                 16    0.458  70.364   1.122   0.003   0.718   0.279
E S  4YFDA                 17    0.462  54.158   0.715   0.004   0.197   0.799
E G  4YFDA                 18    0.707  55.649  -0.632   0.005   0.015   0.979
E G  4YFDA                 19    0.374  29.426   1.101   0.004   0.085   0.910
E V  4YFDA                 20    0.533  81.845   1.385   0.004   0.616   0.381
B A  4YFDA                 21    0.036   3.989   0.233   0.001   0.959   0.040
E S  4YFDA                 22    0.351  41.114   2.012   0.001   0.959   0.040
B F  4YFDA                 23    0.038   7.687   0.539   0.001   0.959   0.040
E I  4YFDA                 24    0.346  64.065   1.959   0.001   0.959   0.040
B C  4YFDA                 25    0.018   2.457   1.186   0.001   0.959   0.040
E Q  4YFDA                 26    0.378  67.457   1.785   0.001   0.959   0.040
B A  4YFDA                 27    0.047   5.212  -0.479   0.001   0.959   0.040
E T  4YFDA                 28    0.493  68.379   0.700   0.002   0.816   0.182
E G  4YFDA                 29    0.369  29.064  -1.485   0.004   0.420   0.576
E D  4YFDA                 30    0.467  67.295   0.286   0.004   0.197   0.799
E P  4YFDA                 31    0.272  38.611  -0.838   0.004   0.138   0.858
E R  4YFDA                 32    0.545 124.736   0.724   0.004   0.138   0.858
B P  4YFDA                 33    0.078  11.082  -0.434   0.004   0.514   0.481
E K  4YFDA                 34    0.354  72.797   1.270   0.001   0.900   0.099
B I  4YFDA                 35    0.031   5.809   0.232   0.001   0.959   0.040
B V  4YFDA                 36    0.240  36.827   1.897   0.006   0.962   0.032
B W  4YFDA                 37    0.030   7.215   0.426   0.006   0.962   0.032
B N  4YFDA                 38    0.228  33.394   1.136   0.001   0.959   0.040
B K  4YFDA                 39    0.218  44.760   1.089   0.002   0.816   0.182
E K  4YFDA                 40    0.539 110.831   0.006   0.005   0.045   0.951
E G  4YFDA                 41    0.716  56.326  -1.445   0.005   0.015   0.979
E K  4YFDA                 42    0.493 101.451   0.603   0.003   0.718   0.279
E K  4YFDA                 43    0.509 104.681   1.203   0.002   0.816   0.182
B V  4YFDA                 44    0.058   8.838   0.181   0.022   0.552   0.426
E S  4YFDA                 45    0.482  56.479   0.851   0.005   0.262   0.733
E N  4YFDA                 46    0.620  90.753  -0.858   0.004   0.085   0.910
E Q  4YFDA                 47    0.639 114.215  -1.873   0.005   0.015   0.979
E R  4YFDA                 48    0.590 135.202  -1.741   0.005   0.045   0.951
E F  4YFDA                 49    0.380  76.306  -1.127   0.004   0.420   0.576
E E  4YFDA                 50    0.291  50.820  -0.043   0.002   0.816   0.182
B V  4YFDA                 51    0.235  36.104  -0.281   0.001   0.959   0.040
B I  4YFDA                 52    0.178  32.912  -0.463   0.001   0.959   0.040
B E  4YFDA                 53    0.239  41.753  -0.836   0.001   0.959   0.040
B F  4YFDA                 54    0.221  44.415  -0.321   0.001   0.900   0.099
E D  4YFDA                 55    0.541  77.944  -0.973   0.004   0.616   0.381
E D  4YFDA                 56    0.594  85.653  -1.803   0.004   0.138   0.858
E G  4YFDA                 57    0.543  42.750  -2.431   0.005   0.015   0.979
E S  4YFDA                 58    0.517  60.604  -1.036   0.004   0.138   0.858
E G  4YFDA                 59    0.274  21.595  -0.065   0.004   0.616   0.381
B S  4YFDA                 60    0.080   9.423  -0.435   0.002   0.816   0.182
B V  4YFDA                 61    0.140  21.457   0.574   0.001   0.900   0.099
B L  4YFDA                 62    0.022   4.101   0.687   0.001   0.959   0.040
B R  4YFDA                 63    0.212  48.479   1.564   0.001   0.959   0.040
B I  4YFDA                 64    0.029   5.328   0.183   0.001   0.900   0.099
E Q  4YFDA                 65    0.397  70.922   1.303   0.003   0.718   0.279
E P  4YFDA                 66    0.503  71.319  -0.036   0.004   0.616   0.381
B L  4YFDA                 67    0.136  24.828  -0.017   0.021   0.451   0.528
E R  4YFDA                 68    0.532 121.782  -0.136   0.005   0.336   0.660
E T  4YFDA                 69    0.412  57.103   0.018   0.005   0.336   0.660
E P  4YFDA                 70    0.566  80.315  -0.604   0.004   0.138   0.858
E R  4YFDA                 71    0.585 134.011  -0.964   0.004   0.085   0.910
B D  4YFDA                 72    0.192  27.653  -1.274   0.004   0.138   0.858
E E  4YFDA                 73    0.442  77.235  -0.490   0.004   0.138   0.858
B A  4YFDA                 74    0.101  11.163  -1.447   0.004   0.616   0.381
E I  4YFDA                 75    0.357  66.064   1.860   0.001   0.900   0.099
B Y  4YFDA                 76    0.035   7.458   0.489   0.001   0.959   0.040
B E  4YFDA                 77    0.216  37.700   2.029   0.001   0.959   0.040
B C  4YFDA                 78    0.013   1.783   1.693   0.001   0.959   0.040
E V  4YFDA                 79    0.235  36.043   2.079   0.001   0.959   0.040
B A  4YFDA                 80    0.025   2.766   0.433   0.001   0.959   0.040
E S  4YFDA                 81    0.355  41.571   1.073   0.001   0.959   0.040
B N  4YFDA                 82    0.114  16.631  -0.350   0.004   0.616   0.381
E N  4YFDA                 83    0.640  93.637   0.022   0.004   0.085   0.910
E V  4YFDA                 84    0.507  77.849  -1.116   0.004   0.138   0.858
E G  4YFDA                 85    0.443  34.880  -1.104   0.005   0.336   0.660
E E  4YFDA                 86    0.556  97.168  -0.506   0.004   0.616   0.381
E I  4YFDA                 87    0.313  57.942  -0.290   0.002   0.816   0.182
E S  4YFDA                 88    0.463  54.264  -0.047   0.001   0.900   0.099
B V  4YFDA                 89    0.169  26.006   0.673   0.001   0.900   0.099
E S  4YFDA                 90    0.422  49.447   0.672   0.001   0.959   0.040
B T  4YFDA                 91    0.040   5.493  -0.197   0.001   0.959   0.040
E R  4YFDA                 92    0.276  63.158   1.499   0.001   0.959   0.040
B L  4YFDA                 93    0.018   3.277   1.239   0.001   0.959   0.040
B T  4YFDA                 94    0.210  29.099   1.790   0.001   0.959   0.040
B V  4YFDA                 95    0.023   3.550   0.986   0.001   0.959   0.040
E L  4YFDA                 96    0.261  47.862   0.980   0.001   0.900   0.099
B R  4YFDA                 97    0.200  45.846   0.519   0.003   0.718   0.279
E E  4YFDA                 98    0.310  54.174   0.196   0.020   0.205   0.775
E D  4YFDA                 99    0.474  68.275  -1.077   0.018   0.088   0.893
E Q  4YFDA                100    0.603 107.660  -1.145   0.019   0.141   0.840
E I  4YFDA                101    0.319  59.089  -1.214   0.019   0.141   0.840
E P  4YFDA                102    0.474  67.204  -1.574   0.018   0.088   0.893
E R  4YFDA                103    0.541 123.866  -1.959   0.019   0.141   0.840
E G  4YFDA                104    0.386  30.355  -1.122   0.019   0.141   0.840
E F  4YFDA                105    0.332  66.612  -0.432   0.018   0.047   0.935
B P  4YFDA                106    0.172  24.336  -1.657   0.019   0.141   0.840
E T  4YFDA                107    0.439  60.903   0.059   0.004   0.616   0.381
B I  4YFDA                108    0.109  20.183  -0.867   0.002   0.816   0.182
E D  4YFDA                109    0.376  54.210  -0.382   0.021   0.756   0.223
E M  4YFDA                110    0.346  69.215  -0.468   0.004   0.514   0.481
E G  4YFDA                111    0.492  38.689  -0.272   0.004   0.138   0.858
B P  4YFDA                112    0.297  42.116  -1.533   0.005   0.045   0.951
E Q  4YFDA                113    0.591 105.606  -0.016   0.005   0.262   0.733
E L  4YFDA                114    0.511  93.546  -0.510   0.003   0.718   0.279
B K  4YFDA                115    0.184  37.808   1.381   0.001   0.900   0.099
E V  4YFDA                116    0.424  65.184   1.021   0.001   0.959   0.040
B V  4YFDA                117    0.069  10.590   0.168   0.001   0.959   0.040
E E  4YFDA                118    0.473  82.616   1.116   0.002   0.816   0.182
E R  4YFDA                119    0.420  96.066   0.508   0.005   0.262   0.733
E T  4YFDA                120    0.688  95.384  -0.518   0.005   0.015   0.979
E R  4YFDA                121    0.508 116.332   1.160   0.004   0.085   0.910
E T  4YFDA                122    0.543  75.314   1.437   0.004   0.616   0.381
B A  4YFDA                123    0.043   4.717   0.317   0.001   0.959   0.040
E T  4YFDA                124    0.338  46.936   1.828   0.001   0.959   0.040
B M  4YFDA                125    0.042   8.464   0.566   0.001   0.959   0.040
E L  4YFDA                126    0.364  66.685   1.577   0.001   0.959   0.040
B C  4YFDA                127    0.019   2.710   1.052   0.001   0.959   0.040
E A  4YFDA                128    0.412  45.380   1.471   0.001   0.959   0.040
B A  4YFDA                129    0.057   6.325  -0.757   0.001   0.900   0.099
E S  4YFDA                130    0.530  62.139  -0.007   0.003   0.718   0.279
E G  4YFDA                131    0.281  22.146  -1.672   0.005   0.262   0.733
E N  4YFDA                132    0.477  69.847   0.008   0.004   0.138   0.858
B P  4YFDA                133    0.246  34.950  -0.704   0.004   0.138   0.858
E D  4YFDA                134    0.511  73.621   0.949   0.004   0.138   0.858
B P  4YFDA                135    0.061   8.670  -0.241   0.004   0.514   0.481
E E  4YFDA                136    0.387  67.679   1.210   0.001   0.900   0.099
B I  4YFDA                137    0.039   7.289   0.310   0.001   0.959   0.040
E T  4YFDA                138    0.247  34.314   1.900   0.001   0.959   0.040
B W  4YFDA                139    0.030   7.095   0.555   0.001   0.959   0.040
B F  4YFDA                140    0.228  45.780   1.127   0.001   0.959   0.040
B K  4YFDA                141    0.210  43.238   0.851   0.002   0.816   0.182
E D  4YFDA                142    0.591  85.178  -0.639   0.018   0.019   0.964
E F  4YFDA                143    0.691 138.724  -2.307   0.018   0.019   0.964
E L  4YFDA                144    0.538  98.544   0.009   0.003   0.718   0.279
E P  4YFDA                145    0.479  67.970   0.045   0.003   0.718   0.279
B V  4YFDA                146    0.099  15.293  -0.161   0.022   0.552   0.426
E D  4YFDA                147    0.426  61.459  -0.128   0.022   0.359   0.619
E T  4YFDA                148    0.370  51.388  -2.071   0.020   0.205   0.775
E S  4YFDA                149    0.446  52.213  -1.948   0.018   0.047   0.935
E N  4YFDA                150    0.542  79.349  -2.452   0.018   0.047   0.935
E N  4YFDA                151    0.513  75.147  -1.410   0.019   0.141   0.840
E N  4YFDA                152    0.484  70.843  -1.999   0.020   0.205   0.775
B G  4YFDA                153    0.304  23.948  -1.594   0.022   0.359   0.619
E R  4YFDA                154    0.333  76.257  -1.092   0.023   0.655   0.322
B I  4YFDA                155    0.202  37.351  -0.605   0.021   0.756   0.223
E K  4YFDA                156    0.408  83.843  -0.796   0.021   0.756   0.223
E Q  4YFDA                157    0.346  61.742  -1.129   0.022   0.552   0.426
E L  4YFDA                158    0.353  64.653  -1.307   0.021   0.451   0.528
E R  4YFDA                159    0.466 106.645  -1.323   0.022   0.359   0.619
E S  4YFDA                160    0.501  58.741  -1.460   0.021   0.451   0.528
E E  4YFDA                161    0.383  66.963  -1.345   0.022   0.552   0.426
E S  4YFDA                162    0.404  47.396  -1.251   0.023   0.655   0.322
B I  4YFDA                163    0.252  46.564  -1.312   0.021   0.756   0.223
E G  4YFDA                164    0.368  28.922  -1.499   0.021   0.756   0.223
E G  4YFDA                165    0.388  30.520  -1.297   0.003   0.718   0.279
E T  4YFDA                166    0.431  59.780  -1.541   0.004   0.616   0.381
E P  4YFDA                167    0.462  65.586  -1.481   0.005   0.262   0.733
E I  4YFDA                168    0.522  96.478  -1.529   0.005   0.262   0.733
E R  4YFDA                169    0.457 104.745  -0.566   0.005   0.336   0.660
B G  4YFDA                170    0.088   6.894  -0.742   0.004   0.514   0.481
B A  4YFDA                171    0.217  23.935   0.633   0.001   0.900   0.099
B L  4YFDA                172    0.020   3.625   0.983   0.001   0.959   0.040
E Q  4YFDA                173    0.234  41.846   1.787   0.001   0.959   0.040
B I  4YFDA                174    0.020   3.774   0.902   0.001   0.959   0.040
E E  4YFDA                175    0.418  73.042   1.719   0.002   0.816   0.182
E Q  4YFDA                176    0.513  91.675   0.522   0.004   0.616   0.381
B S  4YFDA                177    0.078   9.118  -0.047   0.005   0.262   0.733
E E  4YFDA                178    0.465  81.253   0.829   0.005   0.336   0.660
E E  4YFDA                179    0.557  97.308   0.277   0.005   0.262   0.733
E S  4YFDA                180    0.622  72.934  -0.293   0.004   0.085   0.910
B D  4YFDA                181    0.215  30.967  -1.090   0.005   0.045   0.951
E Q  4YFDA                182    0.465  83.049  -0.445   0.004   0.197   0.799
B G  4YFDA                183    0.116   9.137  -1.762   0.002   0.816   0.182
E K  4YFDA                184    0.385  79.236   1.876   0.001   0.959   0.040
B Y  4YFDA                185    0.037   7.992   0.582   0.001   0.959   0.040
B E  4YFDA                186    0.206  36.006   2.008   0.001   0.959   0.040
B C  4YFDA                187    0.011   1.572   1.835   0.001   0.959   0.040
B V  4YFDA                188    0.197  30.233   1.976   0.001   0.959   0.040
B A  4YFDA                189    0.024   2.601   0.507   0.001   0.959   0.040
E T  4YFDA                190    0.306  42.498   0.437   0.001   0.900   0.099
B N  4YFDA                191    0.072  10.585  -1.142   0.004   0.514   0.481
E S  4YFDA                192    0.611  71.656  -0.074   0.018   0.088   0.893
E A  4YFDA                193    0.399  43.948  -1.017   0.018   0.088   0.893
E G  4YFDA                194    0.369  29.080  -1.507   0.004   0.138   0.858
E T  4YFDA                195    0.564  78.227  -1.203   0.019   0.141   0.840
E R  4YFDA                196    0.485 110.996  -1.310   0.021   0.279   0.699
E Y  4YFDA                197    0.417  89.027  -0.692   0.005   0.336   0.660
E S  4YFDA                198    0.381  44.688  -1.132   0.004   0.420   0.576
E A  4YFDA                199    0.358  39.419  -0.337   0.004   0.616   0.381
E P  4YFDA                200    0.337  47.863  -0.367   0.003   0.718   0.279
B A  4YFDA                201    0.091  10.006  -0.519   0.001   0.900   0.099
E N  4YFDA                202    0.301  44.125   0.691   0.001   0.900   0.099
B L  4YFDA                203    0.062  11.389   0.025   0.001   0.959   0.040
E Y  4YFDA                204    0.261  55.797   0.512   0.001   0.959   0.040
B V  4YFDA                205    0.115  17.675  -0.537   0.011   0.918   0.071
E R  4YFDA                206    0.360  82.348   0.067   0.023   0.655   0.322
E E  4YFDA                207    0.388  67.749  -0.803   0.021   0.451   0.528
B L  4YFDA                208    0.270  49.455  -0.360   0.021   0.279   0.699
E R  4YFDA                209    0.549 125.652  -0.797   0.021   0.279   0.699
E E  4YFDA                210    0.402  70.247  -0.426   0.021   0.279   0.699
E V  4YFDA                211    0.334  51.290  -0.935   0.022   0.359   0.619
E R  4YFDA                212    0.390  89.196  -0.809   0.022   0.359   0.619
E R  4YFDA                213    0.574 131.560  -0.711   0.022   0.359   0.619
E V  4YFDA                214    0.314  48.246  -0.540   0.019   0.141   0.840
E P  4YFDA                215    0.315  44.698  -0.416   0.018   0.047   0.935
B P  4YFDA                216    0.166  23.626  -1.814   0.020   0.205   0.775
E R  4YFDA                217    0.409  93.661   0.600   0.023   0.655   0.322
B F  4YFDA                218    0.118  23.622  -1.432   0.021   0.756   0.223
E S  4YFDA                219    0.361  42.309  -0.482   0.023   0.655   0.322
E I  4YFDA                220    0.408  75.406  -0.407   0.021   0.451   0.528
E P  4YFDA                221    0.454  64.394  -0.075   0.004   0.138   0.858
E P  4YFDA                222    0.326  46.274  -1.695   0.018   0.047   0.935
E T  4YFDA                223    0.572  79.406  -0.225   0.021   0.279   0.699
E N  4YFDA                224    0.466  68.252  -0.657   0.004   0.514   0.481
B H  4YFDA                225    0.193  35.107   0.725   0.003   0.718   0.279
E E  4YFDA                226    0.436  76.222   0.465   0.001   0.900   0.099
B I  4YFDA                227    0.062  11.470  -0.179   0.001   0.900   0.099
E M  4YFDA                228    0.408  81.741   0.769   0.002   0.816   0.182
E P  4YFDA                229    0.449  63.685   0.090   0.004   0.420   0.576
E G  4YFDA                230    0.644  50.643  -1.289   0.005   0.045   0.951
E G  4YFDA                231    0.445  35.045  -0.034   0.004   0.085   0.910
E S  4YFDA                232    0.527  61.776   1.019   0.003   0.718   0.279
B V  4YFDA                233    0.047   7.270   0.187   0.001   0.900   0.099
E N  4YFDA                234    0.363  53.085   1.531   0.001   0.959   0.040
B I  4YFDA                235    0.056  10.286   1.031   0.001   0.959   0.040
E T  4YFDA                236    0.369  51.139   1.280   0.001   0.959   0.040
B C  4YFDA                237    0.027   3.861   0.331   0.001   0.959   0.040
E V  4YFDA                238    0.398  61.096   1.377   0.001   0.959   0.040
B A  4YFDA                239    0.045   4.981  -1.018   0.001   0.959   0.040
E V  4YFDA                240    0.496  76.220   0.017   0.002   0.816   0.182
B G  4YFDA                241    0.207  16.283  -1.975   0.004   0.420   0.576
E S  4YFDA                242    0.466  54.603  -0.117   0.004   0.197   0.799
E P  4YFDA                243    0.278  39.505  -0.662   0.004   0.138   0.858
E M  4YFDA                244    0.474  94.847   0.391   0.005   0.262   0.733
B P  4YFDA                245    0.085  12.104  -0.626   0.004   0.514   0.481
E Y  4YFDA                246    0.329  70.329   0.542   0.001   0.900   0.099
B V  4YFDA                247    0.036   5.456   0.259   0.001   0.959   0.040
B K  4YFDA                248    0.232  47.764   1.457   0.001   0.959   0.040
B W  4YFDA                249    0.033   7.912  -0.276   0.006   0.962   0.032
B M  4YFDA                250    0.220  44.002   0.576   0.001   0.900   0.099
B L  4YFDA                251    0.226  41.417   0.551   0.003   0.718   0.279
E G  4YFDA                252    0.551  43.332  -0.622   0.005   0.045   0.951
E A  4YFDA                253    0.686  75.630  -1.849   0.018   0.019   0.964
E E  4YFDA                254    0.523  91.386  -0.304   0.004   0.616   0.381
E D  4YFDA                255    0.541  78.001   0.740   0.003   0.718   0.279
B L  4YFDA                256    0.062  11.425  -0.471   0.022   0.552   0.426
E T  4YFDA                257    0.417  57.824   0.585   0.005   0.262   0.733
E P  4YFDA                258    0.587  83.239  -1.559   0.004   0.138   0.858
E E  4YFDA                259    0.630 110.113  -1.533   0.018   0.047   0.935
E D  4YFDA                260    0.432  62.280  -1.336   0.005   0.262   0.733
E D  4YFDA                261    0.535  77.151  -0.624   0.003   0.718   0.279
E M  4YFDA                262    0.277  55.348  -0.569   0.003   0.718   0.279
E P  4YFDA                263    0.518  73.504  -0.655   0.004   0.616   0.381
E I  4YFDA                264    0.333  61.661  -1.483   0.004   0.420   0.576
B G  4YFDA                265    0.238  18.715  -1.522   0.005   0.336   0.660
E R  4YFDA                266    0.280  64.189  -0.353   0.004   0.616   0.381
B N  4YFDA                267    0.040   5.900  -0.995   0.001   0.900   0.099
B V  4YFDA                268    0.177  27.174   0.830   0.001   0.959   0.040
B L  4YFDA                269    0.022   3.992   0.941   0.001   0.959   0.040
E E  4YFDA                270    0.371  64.761   1.420   0.001   0.900   0.099
E L  4YFDA                271    0.404  74.046  -0.318   0.003   0.718   0.279
B N  4YFDA                272    0.138  20.218   0.500   0.004   0.420   0.576
E D  4YFDA                273    0.479  68.952   0.107   0.004   0.420   0.576
E V  4YFDA                274    0.433  66.583  -0.111   0.005   0.336   0.660
E R  4YFDA                275    0.593 135.889  -0.266   0.004   0.138   0.858
E Q  4YFDA                276    0.312  55.759  -0.720   0.004   0.085   0.910
E S  4YFDA                277    0.415  48.650  -0.759   0.004   0.197   0.799
B A  4YFDA                278    0.126  13.918  -1.983   0.003   0.718   0.279
E N  4YFDA                279    0.395  57.813   1.758   0.001   0.900   0.099
B Y  4YFDA                280    0.041   8.719   0.488   0.001   0.959   0.040
B T  4YFDA                281    0.238  33.038   1.744   0.001   0.959   0.040
B C  4YFDA                282    0.015   2.134   1.475   0.001   0.959   0.040
B V  4YFDA                283    0.220  33.845   1.752   0.001   0.959   0.040
B A  4YFDA                284    0.027   2.942  -0.042   0.001   0.959   0.040
E M  4YFDA                285    0.408  81.601   0.618   0.001   0.900   0.099
B S  4YFDA                286    0.068   7.958  -0.649   0.004   0.514   0.481
E T  4YFDA                287    0.668  92.652  -0.355   0.004   0.138   0.858
E L  4YFDA                288    0.455  83.384  -0.778   0.004   0.197   0.799
E G  4YFDA                289    0.465  36.580  -1.526   0.005   0.336   0.660
E V  4YFDA                290    0.523  80.339  -0.640   0.004   0.514   0.481
E I  4YFDA                291    0.344  63.714  -0.927   0.003   0.718   0.279
E E  4YFDA                292    0.482  84.223  -0.143   0.002   0.816   0.182
B A  4YFDA                293    0.191  21.092   0.098   0.001   0.900   0.099
E I  4YFDA                294    0.360  66.545   0.582   0.001   0.900   0.099
B A  4YFDA                295    0.038   4.199  -0.520   0.001   0.959   0.040
E Q  4YFDA                296    0.262  46.882   1.268   0.001   0.959   0.040
B I  4YFDA                297    0.028   5.161   0.371   0.001   0.959   0.040
E T  4YFDA                298    0.285  39.529   1.476   0.001   0.900   0.099
B V  4YFDA                299    0.051   7.885  -0.047   0.011   0.918   0.071
E K  4YFDA                300    0.347  71.460   0.583   0.018   0.846   0.136
E A  4YFDA                301    0.349  38.438  -0.511   0.004   0.514   0.481
E L  4YFDA                302    0.320  58.647  -0.119   0.020   0.205   0.775
B P  4YFDA                303    0.277  39.363  -1.044   0.018   0.047   0.935
E K  4YFDA                304    0.656 134.919  -0.707   0.018   0.047   0.935
B P  4YFDA                305    0.260  36.908  -0.769   0.018   0.019   0.964
E P  4YFDA                306    0.293  41.563  -0.964   0.018   0.019   0.964
E G  4YFDA                307    0.597  46.992  -1.453   0.018   0.047   0.935
E T  4YFDA                308    0.596  82.637  -0.612   0.018   0.047   0.935
B P  4YFDA                309    0.237  33.559  -0.900   0.019   0.141   0.840
E V  4YFDA                310    0.551  84.673  -0.343   0.022   0.552   0.426
E V  4YFDA                311    0.344  52.904  -0.562   0.021   0.756   0.223
E T  4YFDA                312    0.340  47.103  -0.688   0.021   0.756   0.223
E E  4YFDA                313    0.563  98.374  -0.567   0.023   0.655   0.322
E S  4YFDA                314    0.386  45.298  -0.543   0.004   0.420   0.576
E T  4YFDA                315    0.563  78.130  -1.150   0.004   0.085   0.910
E A  4YFDA                316    0.569  62.671  -0.931   0.005   0.045   0.951
E T  4YFDA                317    0.465  64.565  -0.009   0.005   0.262   0.733
B S  4YFDA                318    0.151  17.709   0.116   0.002   0.816   0.182
B I  4YFDA                319    0.064  11.877   0.159   0.001   0.900   0.099
B T  4YFDA                320    0.177  24.619   0.725   0.001   0.959   0.040
B L  4YFDA                321    0.067  12.176   0.961   0.001   0.959   0.040
B T  4YFDA                322    0.055   7.670  -0.047   0.001   0.900   0.099
B W  4YFDA                323    0.190  45.623   0.022   0.001   0.900   0.099
B D  4YFDA                324    0.246  35.463  -0.073   0.003   0.718   0.279
E S  4YFDA                325    0.435  50.959  -0.357   0.004   0.197   0.799
E G  4YFDA                326    0.694  54.641  -2.916   0.005   0.045   0.951
E N  4YFDA                327    0.613  89.772  -1.464   0.005   0.015   0.979
E P  4YFDA                328    0.323  45.820  -2.002   0.005   0.015   0.979
E E  4YFDA                329    0.518  90.564  -0.063   0.004   0.085   0.910
B P  4YFDA                330    0.112  15.964  -1.132   0.005   0.336   0.660
B V  4YFDA                331    0.235  36.181   0.265   0.002   0.816   0.182
B S  4YFDA                332    0.071   8.345   0.120   0.001   0.959   0.040
B Y  4YFDA                333    0.173  37.013   1.106   0.001   0.959   0.040
B Y  4YFDA                334    0.035   7.522   0.468   0.001   0.959   0.040
B I  4YFDA                335    0.175  32.449   1.120   0.001   0.959   0.040
B I  4YFDA                336    0.140  25.993   1.136   0.001   0.959   0.040
E Q  4YFDA                337    0.428  76.352  -0.170   0.004   0.616   0.381
E H  4YFDA                338    0.497  90.441  -0.755   0.021   0.279   0.699
E K  4YFDA                339    0.484  99.518   0.117   0.002   0.816   0.182
E P  4YFDA                340    0.552  78.272  -0.212   0.002   0.816   0.182
E K  4YFDA                341    0.266  54.696  -0.621   0.021   0.279   0.699
E N  4YFDA                342    0.540  79.012  -0.628   0.004   0.085   0.910
E S  4YFDA                343    0.630  73.883  -1.520   0.004   0.085   0.910
E E  4YFDA                344    0.679 118.709  -2.219   0.005   0.045   0.951
E E  4YFDA                345    0.551  96.330  -0.956   0.004   0.197   0.799
E P  4YFDA                346    0.351  49.849  -0.399   0.004   0.616   0.381
B Y  4YFDA                347    0.217  46.287   0.281   0.001   0.900   0.099
E K  4YFDA                348    0.303  62.245  -0.219   0.001   0.959   0.040
B E  4YFDA                349    0.226  39.570  -0.450   0.001   0.959   0.040
E I  4YFDA                350    0.335  61.956  -0.832   0.001   0.959   0.040
E D  4YFDA                351    0.308  44.455  -1.151   0.001   0.900   0.099
E G  4YFDA                352    0.484  38.107  -0.931   0.004   0.514   0.481
E I  4YFDA                353    0.460  85.063  -1.552   0.004   0.085   0.910
E A  4YFDA                354    0.706  77.768  -0.956   0.004   0.085   0.910
E T  4YFDA                355    0.517  71.680  -0.598   0.005   0.336   0.660
B T  4YFDA                356    0.143  19.820  -0.241   0.003   0.718   0.279
E R  4YFDA                357    0.283  64.921   0.838   0.001   0.900   0.099
B Y  4YFDA                358    0.039   8.420   0.076   0.001   0.959   0.040
E S  4YFDA                359    0.256  30.027   1.611   0.001   0.959   0.040
B V  4YFDA                360    0.040   6.179  -0.090   0.001   0.959   0.040
E A  4YFDA                361    0.403  44.422   1.205   0.001   0.900   0.099
E G  4YFDA                362    0.451  35.517  -0.012   0.003   0.718   0.279
B L  4YFDA                363    0.072  13.202  -0.298   0.005   0.262   0.733
E S  4YFDA                364    0.427  49.998   0.892   0.005   0.262   0.733
E P  4YFDA                365    0.554  78.613   0.170   0.005   0.262   0.733
E Y  4YFDA                366    0.515 110.098  -0.762   0.004   0.085   0.910
B S  4YFDA                367    0.228  26.745  -0.785   0.004   0.138   0.858
E D  4YFDA                368    0.425  61.300   0.321   0.004   0.514   0.481
B Y  4YFDA                369    0.081  17.224  -1.143   0.001   0.900   0.099
E E  4YFDA                370    0.289  50.488   1.993   0.001   0.959   0.040
B F  4YFDA                371    0.027   5.479   0.925   0.001   0.959   0.040
B R  4YFDA                372    0.145  33.297   2.011   0.001   0.959   0.040
B V  4YFDA                373    0.011   1.737   1.866   0.001   0.959   0.040
B V  4YFDA                374    0.176  27.113   1.854   0.001   0.959   0.040
B A  4YFDA                375    0.018   2.028   0.533   0.001   0.959   0.040
B V  4YFDA                376    0.259  39.824   0.545   0.001   0.959   0.040
B N  4YFDA                377    0.081  11.888  -0.412   0.004   0.616   0.381
E N  4YFDA                378    0.584  85.483  -0.175   0.019   0.141   0.840
E I  4YFDA                379    0.398  73.556  -0.719   0.005   0.262   0.733
E G  4YFDA                380    0.285  22.445  -2.057   0.005   0.336   0.660
E R  4YFDA                381    0.567 129.843  -0.686   0.005   0.262   0.733
B G  4YFDA                382    0.275  21.635  -1.197   0.005   0.262   0.733
E P  4YFDA                383    0.573  81.252   0.032   0.005   0.336   0.660
E A  4YFDA                384    0.307  33.831  -0.513   0.004   0.420   0.576
E S  4YFDA                385    0.359  42.075  -0.888   0.004   0.514   0.481
B E  4YFDA                386    0.178  31.166  -0.906   0.002   0.816   0.182
E P  4YFDA                387    0.347  49.253   1.010   0.001   0.900   0.099
B V  4YFDA                388    0.032   4.888   0.045   0.001   0.959   0.040
E L  4YFDA                389    0.307  56.138   1.690   0.001   0.959   0.040
B T  4YFDA                390    0.037   5.118  -0.651   0.001   0.900   0.099
E Q  4YFDA                391    0.319  56.991   1.000   0.001   0.900   0.099
B T  4YFDA                392    0.178  24.730  -0.493   0.004   0.616   0.381
E S  4YFDA                393    0.361  42.344   0.352   0.019   0.141   0.840
E E  4YFDA                394    0.583 101.920  -1.159   0.018   0.019   0.964
E Q  4YFDA                395    0.683 121.912  -1.279   0.018   0.047   0.935
E A  4YFDA                396    0.372  40.972  -1.002   0.018   0.047   0.935
E P  4YFDA                397    0.416  59.002  -0.800   0.018   0.088   0.893
E S  4YFDA                398    0.416  48.779  -1.627   0.018   0.047   0.935
E S  4YFDA                399    0.589  69.043  -1.167   0.018   0.019   0.964
E A  4YFDA                400    0.309  34.052  -0.546   0.018   0.019   0.964
B P  4YFDA                401    0.172  24.421  -2.507   0.018   0.019   0.964
E R  4YFDA                402    0.555 127.049  -0.943   0.019   0.141   0.840
E D  4YFDA                403    0.541  77.958  -0.347   0.004   0.197   0.799
B V  4YFDA                404    0.102  15.708  -0.479   0.004   0.420   0.576
E Q  4YFDA                405    0.543  96.962   0.076   0.002   0.816   0.182
B A  4YFDA                406    0.206  22.657  -0.577   0.001   0.900   0.099
E R  4YFDA                407    0.425  97.440  -0.365   0.001   0.900   0.099
E M  4YFDA                408    0.400  80.040  -1.796   0.003   0.718   0.279
E L  4YFDA                409    0.426  77.946  -1.511   0.004   0.514   0.481
E S  4YFDA                410    0.538  63.030  -1.120   0.004   0.197   0.799
E S  4YFDA                411    0.583  68.374  -0.934   0.005   0.045   0.951
E T  4YFDA                412    0.478  66.285  -0.267   0.004   0.197   0.799
E T  4YFDA                413    0.348  48.295   0.693   0.002   0.816   0.182
B I  4YFDA                414    0.032   5.846  -0.035   0.001   0.900   0.099
E L  4YFDA                415    0.296  54.124   1.482   0.006   0.962   0.032
B V  4YFDA                416    0.027   4.135   0.803   0.006   0.962   0.032
E Q  4YFDA                417    0.276  49.311   1.260   0.011   0.918   0.071
B W  4YFDA                418    0.034   8.081  -0.299   0.001   0.900   0.099
E K  4YFDA                419    0.443  91.207   1.055   0.001   0.900   0.099
B E  4YFDA                420    0.197  34.451  -0.617   0.004   0.616   0.381
E P  4YFDA                421    0.420  59.626  -0.594   0.004   0.138   0.858
E E  4YFDA                422    0.577 100.749  -3.107   0.005   0.045   0.951
E E  4YFDA                423    0.492  85.917  -0.800   0.005   0.045   0.951
E P  4YFDA                424    0.591  83.849  -2.410   0.005   0.015   0.979
E N  4YFDA                425    0.578  84.619  -2.329   0.018   0.019   0.964
B G  4YFDA                426    0.265  20.887  -2.629   0.005   0.045   0.951
E Q  4YFDA                427    0.437  77.977  -0.163   0.004   0.197   0.799
B I  4YFDA                428    0.099  18.315  -1.817   0.004   0.616   0.381
E Q  4YFDA                429    0.247  44.078  -0.223   0.003   0.718   0.279
B G  4YFDA                430    0.177  13.914  -1.066   0.001   0.900   0.099
B Y  4YFDA                431    0.053  11.390   0.240   0.001   0.900   0.099
B R  4YFDA                432    0.142  32.610   0.832   0.001   0.959   0.040
B V  4YFDA                433    0.044   6.778  -0.530   0.001   0.959   0.040
B Y  4YFDA                434    0.123  26.349  -0.192   0.001   0.959   0.040
B Y  4YFDA                435    0.068  14.617  -0.426   0.001   0.959   0.040
B T  4YFDA                436    0.230  31.956   0.568   0.001   0.959   0.040
E M  4YFDA                437    0.391  78.199  -0.582   0.002   0.816   0.182
E D  4YFDA                438    0.447  64.384  -0.501   0.005   0.262   0.733
E P  4YFDA                439    0.696  98.762  -2.301   0.005   0.045   0.951
E T  4YFDA                440    0.580  80.460  -1.488   0.018   0.047   0.935
E Q  4YFDA                441    0.634 113.161  -1.499   0.018   0.088   0.893
E H  4YFDA                442    0.572 104.029  -0.671   0.019   0.141   0.840
E V  4YFDA                443    0.346  53.165  -1.106   0.005   0.262   0.733
E N  4YFDA                444    0.363  53.129  -1.035   0.004   0.514   0.481
E N  4YFDA                445    0.425  62.205  -0.719   0.004   0.616   0.381
B W  4YFDA                446    0.243  58.490  -0.892   0.002   0.816   0.182
E M  4YFDA                447    0.295  59.009  -0.566   0.001   0.900   0.099
E K  4YFDA                448    0.447  91.989  -0.577   0.001   0.959   0.040
E H  4YFDA                449    0.275  50.095  -0.177   0.001   0.959   0.040
E N  4YFDA                450    0.512  75.001  -0.657   0.002   0.816   0.182
E V  4YFDA                451    0.316  48.508  -0.989   0.004   0.420   0.576
E A  4YFDA                452    0.589  64.908  -1.937   0.005   0.045   0.951
E D  4YFDA                453    0.753 108.464  -0.838   0.005   0.015   0.979
E S  4YFDA                454    0.502  58.823  -0.139   0.005   0.262   0.733
B Q  4YFDA                455    0.189  33.773   0.143   0.003   0.718   0.279
E I  4YFDA                456    0.321  59.385   1.126   0.001   0.900   0.099
B T  4YFDA                457    0.037   5.090   0.075   0.001   0.959   0.040
E T  4YFDA                458    0.272  37.754   1.685   0.001   0.959   0.040
B I  4YFDA                459    0.024   4.514   0.394   0.001   0.900   0.099
E G  4YFDA                460    0.461  36.312   1.033   0.003   0.718   0.279
E N  4YFDA                461    0.556  81.442   0.098   0.005   0.262   0.733
B L  4YFDA                462    0.088  16.040   0.306   0.004   0.138   0.858
E V  4YFDA                463    0.524  80.539   1.048   0.004   0.197   0.799
E P  4YFDA                464    0.537  76.144   0.567   0.004   0.138   0.858
E Q  4YFDA                465    0.584 104.356  -0.916   0.005   0.045   0.951
E K  4YFDA                466    0.336  69.156  -0.321   0.004   0.138   0.858
E T  4YFDA                467    0.464  64.398   1.035   0.004   0.616   0.381
B Y  4YFDA                468    0.073  15.707  -0.591   0.001   0.900   0.099
E S  4YFDA                469    0.321  37.598   2.279   0.001   0.959   0.040
B V  4YFDA                470    0.024   3.750   1.050   0.001   0.959   0.040
B K  4YFDA                471    0.177  36.429   2.339   0.001   0.959   0.040
B V  4YFDA                472    0.011   1.675   1.952   0.001   0.959   0.040
B L  4YFDA                473    0.177  32.409   2.144   0.006   0.962   0.032
B A  4YFDA                474    0.018   1.995   0.681   0.001   0.959   0.040
B F  4YFDA                475    0.218  43.692   0.645   0.001   0.959   0.040
B T  4YFDA                476    0.054   7.434  -0.178   0.004   0.616   0.381
E S  4YFDA                477    0.535  62.761  -0.300   0.018   0.088   0.893
E I  4YFDA                478    0.393  72.742  -0.562   0.019   0.141   0.840
B G  4YFDA                479    0.218  17.125  -2.488   0.005   0.336   0.660
E D  4YFDA                480    0.541  77.929  -0.479   0.004   0.197   0.799
B G  4YFDA                481    0.193  15.197  -1.127   0.004   0.138   0.858
E P  4YFDA                482    0.638  90.575   0.123   0.004   0.197   0.799
E L  4YFDA                483    0.386  70.603  -0.648   0.004   0.197   0.799
B S  4YFDA                484    0.259  30.367  -1.148   0.004   0.197   0.799
E S  4YFDA                485    0.343  40.176  -0.572   0.004   0.420   0.576
E D  4YFDA                486    0.431  62.150   1.024   0.002   0.816   0.182
B I  4YFDA                487    0.061  11.359   0.289   0.001   0.959   0.040
E Q  4YFDA                488    0.460  82.245   1.599   0.001   0.959   0.040
B V  4YFDA                489    0.067  10.344  -1.165   0.001   0.959   0.040
E I  4YFDA                490    0.398  73.630   1.991   0.001   0.900   0.099
E T  4YFDA                491    0.454  62.998  -1.182   0.004   0.085   0.910

Explain the output.  Go back.
```

---
